# Patterns of Toll-Like Receptor Expressions and Inflammatory Cytokine Levels and Their Implications in the Progress of Insulin Resistance and Diabetic Nephropathy in Type 2 Diabetic Patients

**DOI:** 10.3389/fphys.2020.609223

**Published:** 2020-12-23

**Authors:** Rofyda H. Aly, Amr E. Ahmed, Walaa G. Hozayen, Alaa Mohamed Rabea, Tarek M. Ali, Ahmad El Askary, Osama M. Ahmed

**Affiliations:** ^1^Biotechnology and Life Sciences Department, Faculty of Postgraduate Studies for Advanced Sciences, Beni-Suef University, Beni-Suef, Egypt; ^2^Biochemistry Department, Faculty of Science, Beni-Suef University, Beni-Suef, Egypt; ^3^Internal Medicine Department, Faculty of Medicine, Beni-Suef University, Beni-Suef, Egypt; ^4^Department of Physiology, College of Medicine, Taif University, Taif, Saudi Arabia; ^5^Department of Physiology, Faculty of Medicine, Beni-Suef University, Beni-Suef, Egypt; ^6^Department of Clinical Laboratory Sciences, College of Applied Medical Sciences, Taif University, Taif, Saudi Arabia; ^7^Department of Medical Biochemistry, Faculty of Medicine (New Damietta), Al Azhar University, Cairo, Egypt; ^8^Physiology Division, Zoology Department, Faculty of Science, Beni-Suef University, Beni-Suef, Egypt

**Keywords:** diabetes mellitus, kidney failure, Toll-like receptors, inflammatory cytokines, insulin resistance

## Abstract

**Background:** Diabetic nephropathy (DNP) is a type 2 diabetes mellitus (T2DM) chronic complication, which is the largest single cause of end-stage kidney disease. There is an increasing evidence of the role of inflammation and Toll-like receptors (TLRs) as part of innate immune system in its development and progression. In addition, Toll-like receptor 2 (TLR2) and Toll-like receptor 4 (TLR4) downward signaling causes the production of proinflammatory cytokines, which can induce insulin (INS) resistance in T2DM.

**Objective:** The goal of this study was to estimate the expression of TLRs (TLR2 and TLR4) in relation to inflammation and INS resistance in nephrotic type 2 diabetic patients with or without renal failure and to discuss the role of these TLRs in DNP progression.

**Patients and Methods:** In this study, blood samples were obtained from type 2 diabetic patients with or without renal failure, and patients with non-diabetic renal failure were compared to healthy controls. All participants were tested for analysis of fasting plasma glucose and serum insulin, kidney function tests, C-reactive protein (CRP), and proinflammatory cytokines, including tumor necrosis factor alpha (TNF-α), interferon gamma (IFN-γ), and interleukin 6 (IL-6) as well as expression of TLR2 and TLR4 in peripheral blood (PB). Statistical analysis of data was done by using SPSS.

**Results:** Diabetic patients with renal failure exhibited significant increase in TLR2, TLR4 mRNA expression in PB in comparison with normal subjects, diabetic patients without renal failure and non-diabetic patients with renal failure. Both diabetic patients with or without kidney failure and non-diabetic patients with renal failure had increased TLR2 and TLR4 mRNA expression in association with increased levels of proinflammatory cytokines (TNF-α, IFN-γ, and IL-6) compared to normal subjects. The diabetic patients with kidney failure exhibited the highest elevation of TLRs, Th1 cytokines and CRP in association the highest record of insulin resistance.

**Conclusion:** Toll-like receptor 2 and Toll-like receptor 4 increased expression and Th2 cytokines may have an important role in the progression of DNP and deteriorations in insulin resistance in type 2 diabetic patients. Therefore, TLR2 and TLR4 may be a promising therapeutic target to prevent or retard DNP in type 2 diabetic patients.

## Introduction

**Figure 1:**
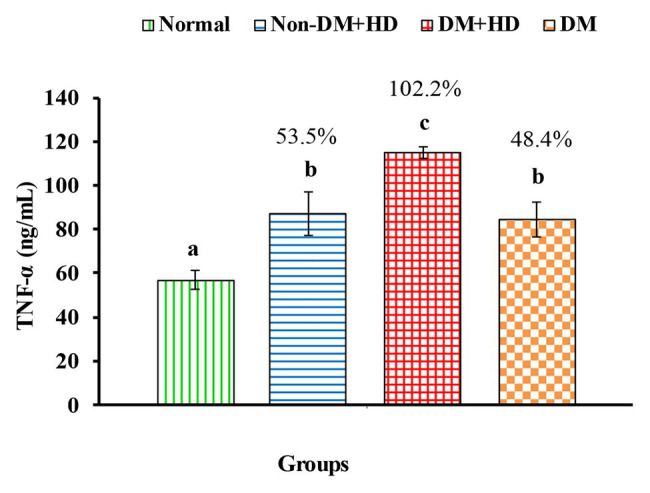
The levels of tumor necrosis factor alpha [TNF-α (ng/mL)] in serum of non-diabetic and diabetic patients with or without kidney failure. Means, which have different superscript letters, are significantly different at *p* < 0.05. Percentage changes were calculated in comparison with normal group. Value of *p*: 0.003.

Diabetes mellitus (DM) is a chronic metabolic endocrinal disease, which is characterized by elevation in blood glucose level and is associated with multiple complications. Type 2 DM (T2DM) is the most common form of DM (Arababadi et al., 2009). T2DM becomes a major health burden for chronic illness in Africa (Badr et al., 2019). Many complications arising from having either T1DM or T2DM affect vital organs in the body like kidneys (nephropathy; Rosa and Ravi, 2012). Diabetic nephropathy (DNP) is one of the most medical concerns of DM because in recent years, it has become the leading cause for the end-stage kidney disease (Ruiz-Ortega et al., 2020; Zhang et al., 2020). DNP develops to end-stage renal disease *via* a number of stages, including normal albuminuria, incipient DNP, micro albuminuria, and finally end-stage renal disease (Singh et al., 2014). The incidence of DNP is affected by the hemodynamic disorders, genetic factors, and disorders of biochemical metabolism (Liu et al., 2010). Emerging evidence states that inflammatory mechanisms play a crucial role in disease progression and pathogenesis (Pérez-Morales et al., 2019; Rayego-Mateos et al., 2020). Innate immune system and low-grade inflammation are significantly associated to the incidence and severity of T2DM and its complications (Navarro-Gonzalez and Mora-Fernandez, 2008; Pashkow, 2011). Inflammation can be a key factor triggered by the biochemical, metabolic, and hemodynamic perturbations that exist in the diabetic kidney (Lim and Tesch, 2012). Proinflammatory cytokines produced by T helper cells (Th1) such as interleukin-1 (IL-1), tumor necrosis factor-α (TNF-α), and interferon-γ (IFN-γ) can induce resident renal cells such as tubular epithelial cells, mesangial cells, and podocytes to produce chemokines (Lim and Tesch, 2012). Such chemokines function as “chemoattractant” molecules, playing a key role in inflammatory cell recruitment, migration, and interaction, as well as in cellular adhesion, differentiation, and tissue damage in the setting of DNP (Ruster and Wolf, 2008; Pérez-Morales et al., 2019). During long standing hyperglycemic state in DM, glucose interacts with the plasma proteins through a non-enzymatic process (Maillard reaction; Helou et al., 2014). In this reaction, by which glycation alters the cell functions include denaturation and functional decline of the target protein and lipid, organopathy due to accumulation of advanced glycation end products (AGEs) in tissue (Yonekura et al., 2005). AGEs are stimulators for the production of chemokine (Lim and Tesch, 2012) and play a causal role in both the microvascular and macrovascular diabetes complications, including retinopathy, neuropathy, nephropathy, also atherosclerosis (McCance et al., 1993; Beisswenger et al., 1995; Xu et al., 2018). Inflammatory chemokines are related to proinflammatory mechanisms and induce leukocyte recruitment to damaged tissues (Chang and Chen, 2020). In case of nephropathy, chronic exposure to diabetic substrates damages renal cells, resulting in injury or cell death and the release of intracellular damage-associated molecular patterns (DAMPs) into the extracellular space (Figure 1). This signal is recognized by pattern recognition receptors (PRRs) such as Toll-like receptors (TLRs; Tang and Yiu, 2020).

Toll-like receptors are transmembrane proteins that transfer the antigen recognition information from outside to inside of the cell as a factor in the immune reaction (Zhu et al., 2011; Mishra and Pathak, 2019). Activation of the innate immune system *via* TLRs is implicated in the pathogenesis of insulin (INS) resistance, DM, and atherosclerosis (Shi et al., 2006; Wong et al., 2007; Curtiss and Tobias, 2009). TLR4 is the main part of immune-mediated and inflammatory reactions and plays a role both in the recognition of lipopolysaccharides and signal transduction of immune-mediated inflammatory reactions (Lepenies et al., 2011). TLR2 and/or TLR4 might be a molecular link between inflammation and DM as they promote tubulointerstitial inflammation during DNP (Devaraj et al., 2009). TLR4 has a major role in renal inflammation and progressive fibrosis in kidney disease (Yiu et al., 2014). Modulating these TLRs could be helpful in preventing complications of DM giving the key role of inflammation in both microvascular and macrovascular complications (Jialal and Kaur, 2012; Badr et al., 2019).

Therefore, this study was conducted to scrutinize the patterns of changes and roles of TLR2 and TLR4 expressions in relation to proinflammatory Th1 cytokine levels and INS resistance in nephrotic diabetic patients with or without kidney failure. The comprehension of such relations in different stages may be beneficial in determining new therapeutic strategies targeting inflammation to prevent and/or retard DNP in T2DM.

## Materials and Methods

### Ethical Approval and Consent

The work in this study was approved by the Ethics Committee of Faculty of Post-graduate studies for Advanced Science, Beni-Suef University, Egypt (Ethical Approval Number: 3/019).

### Study Design and Subjects

The target population of this study comprises DNP patients with or without renal impairment either on hemodialysis (HD) or not, from Beni-Suef University Hospital and other local hospitals. The sample was classified into four groups (each of 20 patients). Group 1 (Normal) includes healthy individuals. Group 2 (non-DM + HD) consists of non-diabetic patients that have renal failure and are subjected to HD. Group 3 (DM + HD) consists of diabetic patients that have renal failure and are subjected to HD. Group 4 (DM) consists of diabetic patients that have no renal failure and are not subjected to HD.

### Exclusion Criteria

Patients having autoimmune diseases or recently hospitalized for infectious disease.

### Laboratory Assessment

After obtaining a full history from all participants, 6 mL of the peripheral blood (PB) from all the study participants were obtained aseptically by venipuncture.

Obtained blood samples from overnight-fasted subjects were divided into 2 mL in a plain tube for biochemical studies and detection of proinflammatory cytokines, 2 mL in sodium fluoride tube for the determination of plasma glucose level, and 2 mL in dipotassium ethylenediaminetetraacetic acid (EDTA) for TLR2 and TLR4 expression examination.

### Biochemical Investigations

Plasma glucose level was determined with glucose oxidase, and 4-aminoan using reagent kit purchased from Vitro Scient in Egypt (Trinder, 1969). Serum insulin was determined according to the quantitative sandwich enzyme immunoassay technique by using Human INS ELISA kit (Catalog Number for insulin is MBS704195). Homeostasis Model Assessment-Insulin Resistance (HOMA-IR), Homeostasis Model Assessment-Insulin Senstivity (HOMA-IS), and Homeostasis Model Assessment-beta-cell function (HOMA-β cell function) were evaluated according to Park et al. (2009), Krishnaveni et al. (2010), and Kuang et al. (2015) as follow:

HOMA-IR = fasting insulin level (μIU/mL) × fasting blood glucose (mmol/L)/22.5.

HOMA-IS = 10,000/fasting insulin × fasting glucose.

HOMA-β cell function = [20 × fasting insulin (μIU/mL)] ÷ {[fasting glucose (mg/dL) ÷ 18] − 3.5}.

Serum urea level was detected according to the method modified Berthelot reaction using reagent kit obtained from Vitro Scient in Egypt (Fawcett and Scott, 1960). Serum creatinne level was detected based on Jaffé (1886) reaction without deproteinization using reagent kit obtained from Vitro Scient in Egypt. Serum Uric acid level was determined by using reagent kit obtained from Vitro Scient in Egypt (Gochman and Schmitz, 1971). Level of serum cystatin C, as a good marker of glomerular filtration rate and impairment of kidney clearance, was measured by an enzyme-linked immunosorbent assay (ELISA) kit purchased from MyBioSource, San Diego, California, United States according to manufacturer’s instructions. C-reactive protein (CRP) was determined according to latex agglutination reaction using reagent kit obtained from Vitro Scient in Egypt (Kindmark, 1972; Otsuji et al., 1982). The estimated glomerular filtration rate (eGFR) was calculated using the MDRD equation (Levey et al., 1999, 2006).

### Measurements of Cytokine Levels

Sandwich ELISA was applied for quantitative detection of serum TNF-α, IFN-γ, and IL-6 levels using kits obtained from MyBioSource, San Diego, California, United States; the catalog numbers for these kits were MBS015648, MBS824507, and MBS261259, respectively.

### RNA Extraction

Total RNA was extracted from whole blood by using a Trizol kit obtained from Ambion, Life Technologies, Carlsbad, CA (Chomczynski and Sacchi, 1987; Chomczynski, 1993). Cloned DNA (cDNA) was synthesized by using reagent kit obtained from Applied Biosystems, United States. The total RNA concentration was determined by using nonodrop 2000, the parameters for the integrity of the extracted RNA (ratio A260/280) was about 1.8 and the amount of total RNA used for cDNA synthesis was about 10 μl of total RNA of concentration, from 80 to 300 ng/μl.

### Quantification of TLR2 and TLR4 Expressions

Quantitative real-time PCR technique carried out to detect mRNA expression of TLR2 and TLR4 in PB by using a universal Taqman master mix and a gene expression ready-made assay (Applied Biosystems, United States). A volume of 2.4 μl of cDNA was used as a template in 20 μl reaction containing 10 μl of TaqMan Universal Master Mix II, 1.0 μl of TaqMan Assay and 9.0 μl of cDNA/DNA template + RNase-free water. Holding stage includes two cycles, cycle 1 (50°C/2 min) and cycle 2 (95°C/10 min). Amplification stage includes 40 cycles; each cycle includes (95/15 s and 60/1 min). All data were presented compared with control after normalization to GAPDH (housekeeping gene). The Taqman(R) Gene Expression Assay ID of TLR2 is Hs00610101_m1 and Taqman(R) Gene Expression Assay ID of TLR4 is Hs00152939_m1.

### Statistical Analysis

Gene expression was calculated using ∆ ∆ Ct method. Data were analyzed by using one-way ANOVA and Duncan’s test using the statistical package for the social sciences (IBM SPSS, version 20.0). Results were expressed as mean ± SE. Data of values of *p* > 0.05 were considered not significantly different, while those of values of *p* < 0.05 were significantly different. Correlation analysis was also performed by IBM SPSS, version 20.0 to assess correlations between different variables.

## Results

Demography of the studied groups in Table 1 revealed that group of 20 healthy volunteers of age 47.3 ± 1.45 years consists of 14 (70%) males and six (30%) females were included in this study and served as control (group 1). The group 2 (non-DM + HD) consists of 20 non-diabetic subjects, with kidney failure, of age 52.6 ± 1.45 years and includes 13 (65%) males and seven (35%) females. The group 3 (DM + HD) is composed of 20 diabetic patients with kidney failure; their age was 54.6 ± 2.02 years and consists of 11 (55%) males and nine (45%) females. The group 4 of age 52.0 ± 0.57 years consists of 12 (60%) males and eight (40%) females with DM without kidney failure.

**Table 1:** Demographic data of studied groups.

Variables		Group 1 (Normal)	Group 2 (non-DM + HD)	Group 3 (DM + HD)	Group 4 (DM)
Number of subjects		20	20	20	20
Age (Years)	Mean ± SE	47.3 ± 1.45	52.6 ± 1.45	54.6 ± 2.02	52.0 ± 0.57
Gender	Male	14 (70%)	13 (65%)	11 (55%)	12 (60%)
	Female	6 (30%)	7 (35%)	9 (45%)	8 (40%)

Biochemical analysis and comparison between the studied groups are shown in Table 2. Analysis of fasting plasma glucose level between the four groups indicated that there was a significant difference between group 1 (normal), on one hand, and group 3 (DM + HD) and group 4 (DM) on the other, but there was no significant difference between group 1 (normal) and group 2 (non-DM + HD). Analysis of fasting insulin level between the four groups showed that there was a significant difference between group 1 (normal) and group 3 (DM + HD). The calculated HOMA-IR value showed that there was a significant difference between group 1 (normal) and group 3 (DM + HD). HOMA-IS value exhibited a significant difference between group 1 (normal), on one hand, and group 3 (DM + HD) and group 4 (DM), on the other. HOMA-β Cell Function value showed a significant difference between group 1 (normal) and other groups as well as between group 2 and the two diabetic groups (groups 3 and 4). The serum cystatin C level showed a significant elevation in group 2 (non-DM + HD), group 3 (DM + HD) and group 4 (DM) as compared with normal healthy control; the percentage increases were 211.9, 244.0, and 193.6 respectively. Group 3 (DM + HD) had significantly higher serum cystatin C when compared with group 2 (DM). Analysis between the four groups regarding the serum urea level showed that there was a significant difference between group 1 (normal) and group 2 (non-DM + HD) and group 3 (DM + HD), but there was a non-significant difference between group 1 (normal) and group 4 (DM). Analysis between the four groups regarding the serum creatinine level showed that there was a significant difference between group 1 (normal) and group 2 (non-DM + HD) and group 3 (DM + HD), but there was no a significant difference between group 1 (normal) and group 4 (DM). Serum uric acid level exhibited a significant difference between group 1 (normal) and each of the other three groups. Serum CRP level showed a significant difference between group 1 (normal), group 3 (DM + HD), and group 4 (DM) and there was an increase between group 1 (normal) and group 2 (non-DM + HD). The eGFR showed that there was a significant difference between group 1 (normal) and each of the other three groups, but there was no a significant difference between group 2 (non-DM + HD) and group 3 (DM + HD).

**Table 2:** Biochemical analysis of patients and control groups.

Variables	Group 1 (Normal)	Group 2 (non-DM + HD)	Group 3 (DM + HD)	Group 4 (DM)	*p* value
Plasma glucose (mg/dL) % Change	80.1 ± 1.4^a^ -	94.1 ± 5.8^a^ 17.4	230.6 ± 28.9^b^ 187.9	200.1 ± 21.0^b^ 149.8	0.001^*^ -
Fasting insulin (μIU/mL) % Change	15.8 ± 2.5^a^ -	16.1 ± 1.07^a^ 1.9	23.3 ± 3.3^b^ 47.4	10.8 ± 0.9^a^ ‐ 31.6	0.02^*^
HOMA-IR % Change	3.02 ± 0.53^a^ -	3.9 ± 0.36^a^ 29.1	13.4 ± 1.2^b^ 1038.0	6.2 ± 1.4^a^ 105.2	0.001^*^
HOMA-IS % Change	8.5 ± 1.3^c^ -	6.1 ± 0.5^bc^ ‐ 28.2	1.8 ± 0.1^a^ ‐ 78.8	4.2 ± 0.7^ab^ ‐ 50.6	0.002^*^ -
HOMA-β cell function % Change	388.0 ± 29.3^c^ -	153.0 ± 18.7^b^ ‐ 60.6	50.0 ± 13.0^a^ ‐ 87.1	23.7 ± 2.2^a^ ‐ 93.9	0.001^*^ -
Serum Cystatin C (mg/L)	1.09 ± 0.20^a^ -	3.40 ± 0.70^bc^ 211.9	3.75 ± 0.58^c^ 244.0	3.20 ± 0.23^b^ 193.6	0.000^*^ -
Serum urea (mg/dL) % Change	34.1 ± 1.4^a^ -	117.8 ± 9.1^b^ 245.4	107.1 ± 8.7^b^ 214.0	44.6 ± 5.3^a^ 30.8	0.001^*^ -
Serum creatinine (mg/dL) % Change	0.91 ± 0.06^a^ -	7.9 ± 0.44^b^ 768.1	7.7 ± 0.65^b^ 746.1	1.46 ± 0.11^a^ 42.8	0.001^*^ -
Serum uric acid (mg/dL) % Change	3.2 ± 0.18^a^ -	7.0 ± 0.17^c^ 118.7	7.2 ± 0.29^c^ 125.0	5.06 ± 0.35^b^ 58.1	0.001^*^ -
Serum CRP (mg/L) % Change eGFR (mL/min/1.73m^2^) % Change	1.5 ± 0.24^a^ - 83.8 ± 2.1^c^ -	3.7 ± 0.34^a^ 146.6 10.2 ± 0.34^a^ −73.6	22.0 ± 2.0^c^ 1366.6 6.8 ± 0.55^a^ −91.9	10.6 ± 3.6^b^ 606.6 47.6 ± 3.4^b^ −43.2	0.001* - 0.001^*^ -

Data are expressed as mean ± SE. For each variable, values, which have different superscript letters, are significantly different at *p* < 0.05.

**Figure 2:**
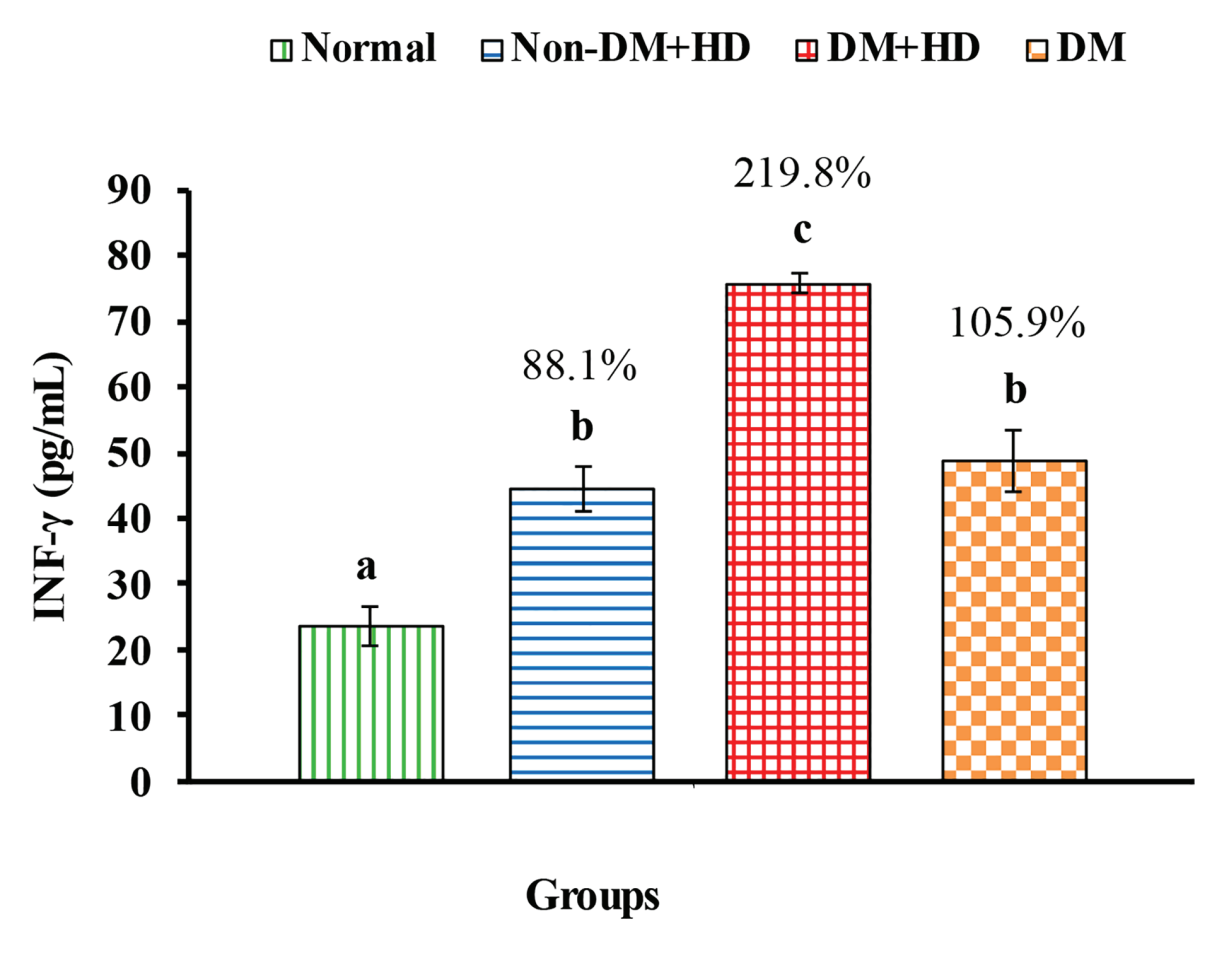
The level of interferon gamma [IFN-γ (pg/mL)] in the blood of non-diabetic and diabetic patients with or without kidney failure. Means, which have different superscript letters, are significantly different at *p* < 0.05. Percentage changes were calculated in comparison with normal group. Value of *p*: 0.001.

**Figure 3:**
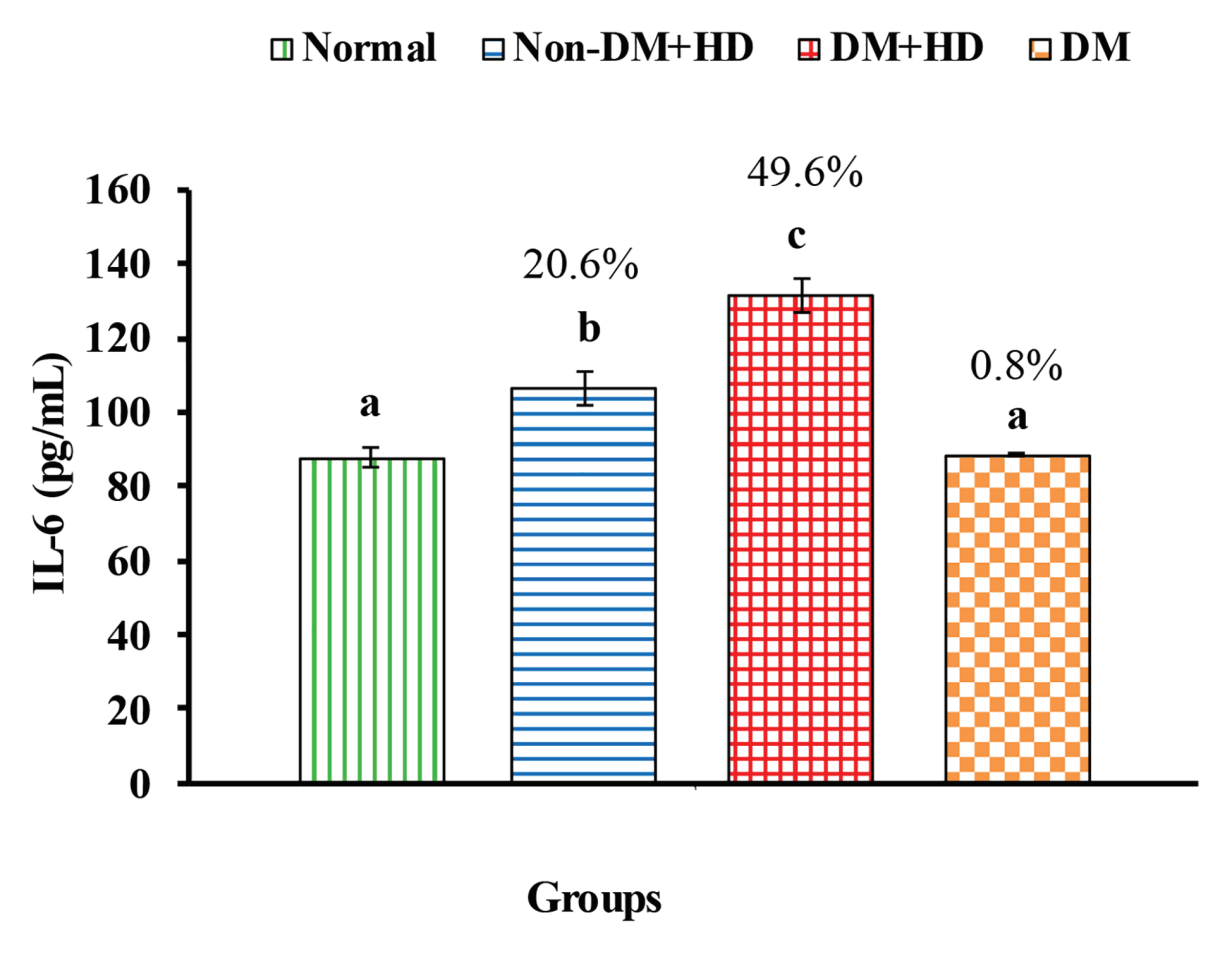
The level of interleukin 6 [IL-6 (pg/mL)] in the blood of non-diabetic and diabetic patients with or without kidney failure. Means, which have different superscript letters, are significantly different at *p* < 0.05. Percentage changes were calculated in comparison with normal group. Value of *p*: 0.001.

Analysis between the four groups regarding the level of TNF-α showed that there was statistically significant difference between group 1 and the other three groups as indicated in Figure 1. The percentages were 53.5, 102.2, and 48.4% when comparing normal group with non-DM + HD, DM + HD and DM groups, respectively. Serum TNF-α level of diabetic patients with kidney failure exhibited a significant increase when compared with that of patients with either DM or kidney failure. Similar to TNF-α, serum IFN-γ level showed a significant difference between group 1 and the other 3 groups as indicated in Figure 2. The percentage changes were 88.1, 219.8, and 105.9%, respectively. Serum IFN-γ level of diabetic patients with kidney failure exhibited a significant increase when compared with that of patients with either DM or kidney failure. IL-6 level showed a significant difference between group 1, on one hand, and groups 2 and 3, on the other, as indicated in Figure 3. The percentage changes were 20.6, 49.6, and 0.8%, respectively for groups 2, 3, and 4 as compared with normal subjects. Serum IL-6 level of diabetic patients with kidney failure exhibited a significant increase when compared with that of patients with either DM or kidney failure.

**Figure 4:**
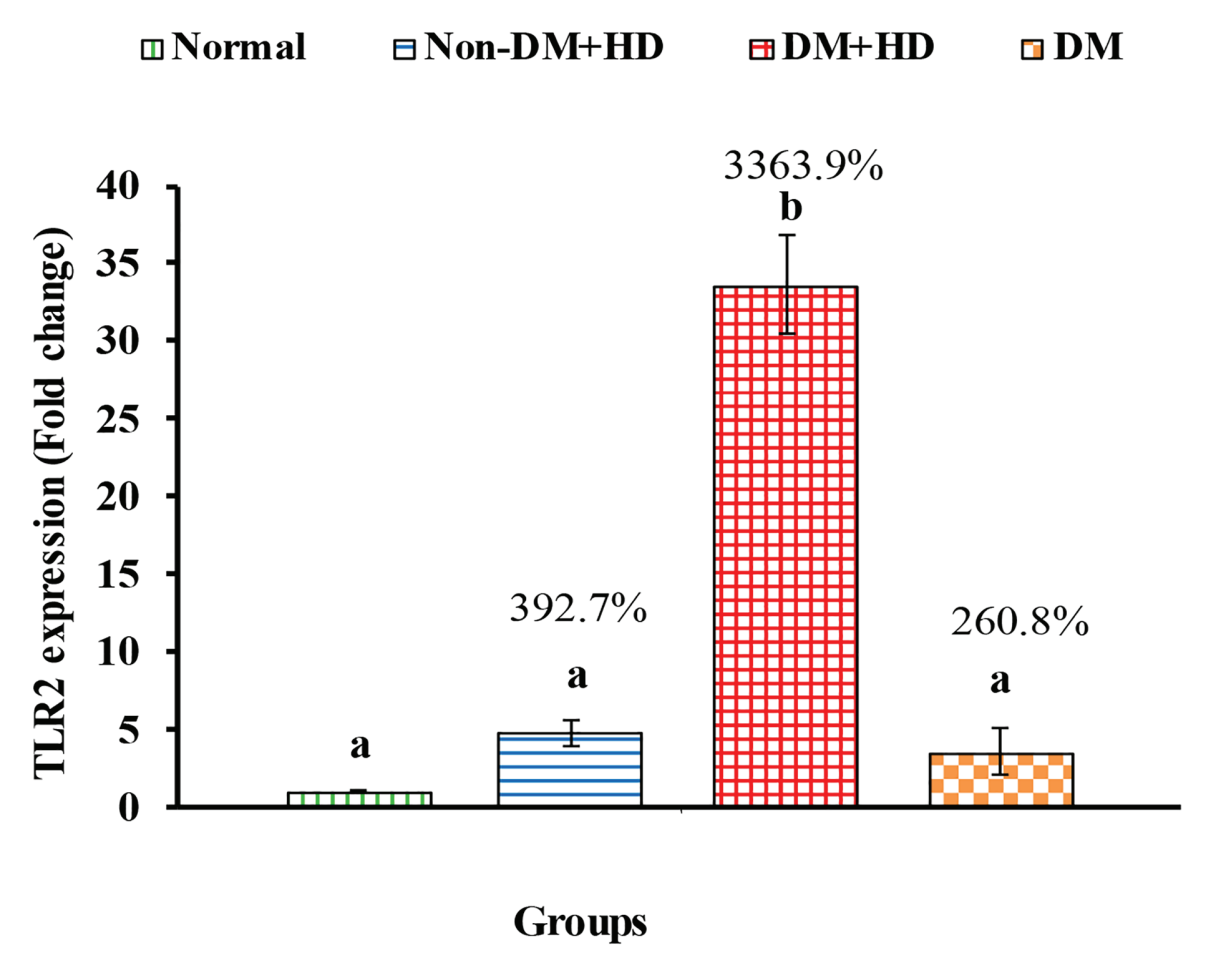
The expression of Toll-like receptor 2 (TLR2) in the blood of non-diabetic and diabetic patients with or without kidney failure. Means, which have different superscript letters, are significantly different at *p* < 0.05. Percentage changes were calculated in comparison with normal group. Value of *p*: 0.001.

TLR2 mRNA expression of exhibited a marked increase in group 2 (Non-DM + HD), group 3 (DM + HD), and group 4 (DM) recording percentage changes of 392.7, 3363.9, and 260.8%, respectively as compared to normal; the most potent effect was attained in group 3 (DM + HD). While the effect was significant in group 3 (DM + HD), it was non-significant in group 2 (Non-DM + HD) and group 4 (DM) as compared with normal. TLR2 mRNA expression was found to be significant in diabetic patients with kidney failure when compared with patients with either DM or kidney failure as indicated in Figure 4.

**Figure 5:**
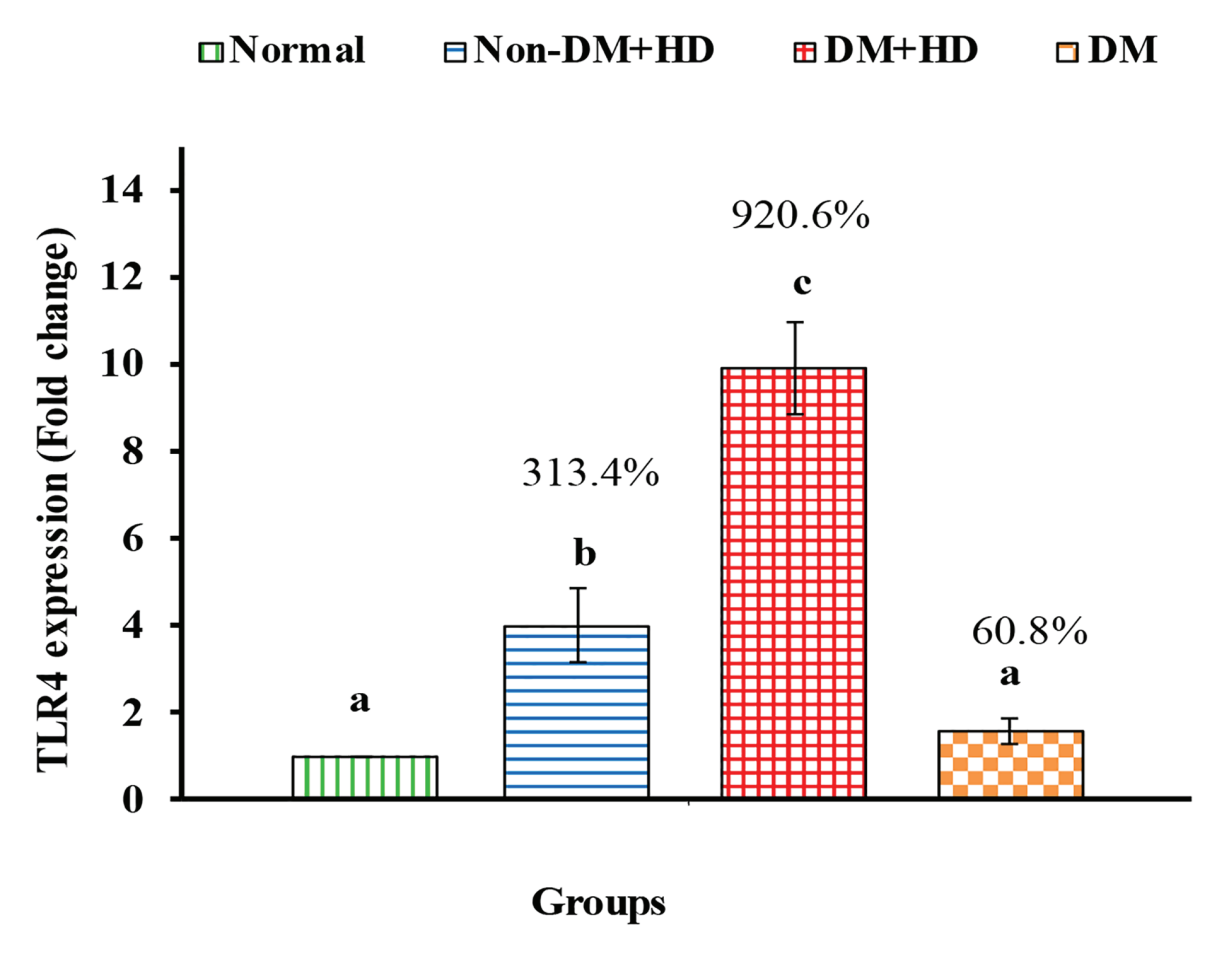
The expression of Toll-like receptor 4 (TLR4) in the blood of non-diabetic and diabetic patients with or without kidney failure. Means, which have different superscript letters, are significantly different at *p* < 0.05. Percentage changes were calculated in comparison with normal group. Value of *p*: 0.001.

TLR4 mRNA expression markedly increased in all patient groups including group 2 (non-DM + HD), group 3 (DM + HD), and group 4 (DM) groups recording percentage changes of3.13.4, 920.6, and 60.8%, respectively as compared to normal; the most potent deleterious effect was attained in DM + HD group. While the effect was significant in groups Non-DM + HD and DM + HD, while it was non-significant in DM group as compared with normal. TLR4 mRNA expression was found to be significant in diabetic patients with kidney failure when compared with patients with either DM or kidney failure (Figure 5).

The correlations between the inflammatory indictor (CRP), inflammatory cytokines, and TLR2 and TLR4 on one hand, and HOMA-IR, HOMIS, and kidney function parameters on the other, were represented in Table 3. The CRP, TNF-α, INF-γ, and IL-6 serum levels as well as TLR2 and TL4 expression have positive significant correlations with HOMA-IR and serum biomarkers of kidney dysfunction and have negative significant correlation with HOMA-IS. Moreover, the TLR2 and TLR4 expressions have positive significant correlations with the serum levels of CRP, TNF-α, INF-γ, and IL-6.

**Table 3:** Correlation analysis between inflammatory mediators, insulin resistance, and kidney function parameters.

		CRP	TNF-α	INF-γ	IL-6	TLR2	TLR4
HOMA-IR	r *p*-value	0.937 0.000	0.741 0.000	0.677 0.000	0.782 0.000	0.818 0.000	0.784 0.000
HOMA-IS	r *p*-value	−0.865 0.000	−0.740 0.000	−0.659 0.000	−0.635 0.01	−0.698 0.000	−0.643 0.01
Cystatin C	r *p*-value	0.646 0.010	0.809 0.000	0.621 0.001	0.611 0.002	0.536 0.007	0.565 0.004
Urea	r *p*-value	0.379 0.068	0.648 0.001	0.727 0.000	0.771 0.000	0.535 0.007	0.676 0.000
Creatinine	r *p*-value	0.431 0.035	0.671 0.000	0.750 0.000	0.816 0.000	0.597 0.002	0.724 0.000
Uric acid	r *p*-value	0.587 0.003	0.787 0.000	0.799 0.000	0.782 0.000	0.615 0.001	0.715 0.000
TLR2	r *p*-value	0.889 0.000	0.726 0.000	0.707 0.000	0.825 0.000	-	0.837 0.000
TLR4	r *p*-value	0.771 0.000	0.692 0.000	0.797 0.000	0.810 0.000	0.837 0.000	-

r: Pearson correlation coefficient; *p* < 0.05 is significant correlation; +: positive correlation; −: negative correlation.

## Discussion

**Figure 6:**
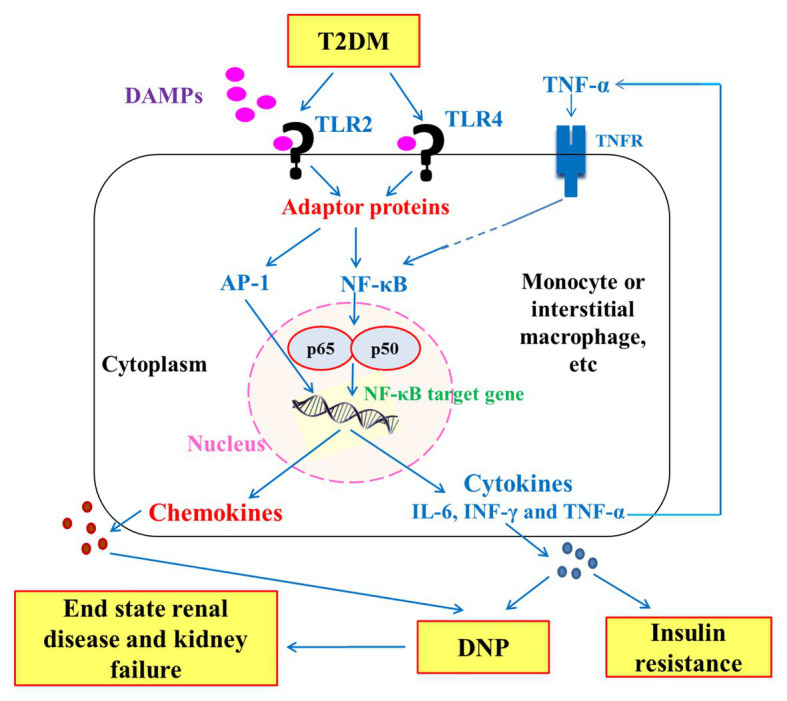
Schematic diagram showing the downward signaling pathways of TLR2 and TLR4 to the release inflammatory cytokines that have an important role in insulin resistance and pathogenesis of diabetic nephropathy (DNP) in type 2 diabetes mellitus (T2DM). DAMPs: Damage-associated molecular patterns; TNFR: tumor necrosis factor receptor; NF-*κ*B: nuclear factor-kappaB; and AP-1: Activator protein 1.

Most patients with T2DM suffer from serious complications of chronic hyperglycemia including nephropathy, retinopathy, neuropathy, and accelerated development of cardiovascular diseases (Grant et al., 2006). DNP is the most important microvascular complication associated with diabetes, and it is one of key factors that lead to end-stage renal disease (White et al., 2005; Yang et al., 2012). Preclinical evidence supported the concept that TLR2 and/or TLR4 are causative in diabetic kidney disease (DKD; Panchapakesan and Pollock, 2018). Network cytokines play a key role in the orientation of the immune responses (Arababadi et al., 2009). TLRs are the only key transmembrane proteins in mammals that transfer the antigen recognition information from the outside to the inside of the cell as an important factor in the immune response (Zhu et al., 2011). Among the TLRs, TLR2 and TLR4 play a crucial role in inflammation and DM pathogenesis (Figure 6) under clinical and experimental conditions (Dasu et al., 2010). TLR2 and TLR4 bind to components of the Gram-positive and Gram-negative bacteria, respectively (Beutler, 2004).

In this study, the serum levels of proinflammatory Th1 cytokines TNF-α, IFN-γ, and IL-6 were higher in patient groups than the control group. Moreover, the serum levels of these cytokines were higher in diabetic patients with kidney failure than in diabetic patients without kidney failure and non-diabetic subjects with kidney failure. These higher levels of these inflammatory cytokines in diabetic patients with kidney failure reflects the important roles of these cytokines and inflammation in the progress of DN and the advanced disease state to reach end-stage renal disease and kidney failure. In parallel with the present study, Nosratabadi et al. (2009) found a significant difference in serum level of IFN-γ in nephrotic diabetic patients and controls subjects and Wong et al. (2007) who reported elevated levels of other inflammatory cytokines like IL-6, IL-18, and TNF-α in DNP. It is worth mentioning here that the highest levels of serum TNF-α, IFN-γ, and IL-6 in diabetic patients with kidney failure were associated with the most deleterious effects on insulin resistance and insulin sensitivity, since the nephrotic diabetic patients with kidney failure have the highest value of HOMA-IR and the lowest value of HOMA-IS as compared with diabetic patients without kidney failure and non-diabetic subjects with kidney failure. Concomitant with these effects on serum TNF-α, IFN-γ, and IL-6 levels, HOMA-IR and HOMA-IS, the CRP (marker of inflammation) was most deleteriously affected in nephrotic diabetic patients without kidney failure. As indicated by correlation analysis in the present study, the CRP, TNF-α, IFN-γ, and IL-6 serum levels have positive significant correlation with HOMA-IR and negative significant correlation with HOMA-IS. Thus, it can be concluded that the worst effect on insulin resistance and insulin sensitivity in type 2 diabetic patients with end-stage renal disease and kidney failure may be secondary to the strongest effects of inflammation.

As indicated in the present study, in the diabetic patients with renal failure, the expression of TLRs was significantly higher than normal group and other groups. The expressions of TLR2 and TLR4 remarkably increased in nephrotic diabetic patients with or without kidney failure and in non-diabetic nephrotic patients (with kidney failure) in comparison with normal subjects. The effect on TLRs expression was highest in diabetic nephrotic patients with end disease state and kidney failure. The higher expression of TLR2 and TLR4 in nephrotic diabetic patients with kidney failure than in nephrotic diabetic patients without kidney failure reflects their important roles in the progress of DNP to reach the end disease state subjected to HD. In support with this elucidation, the present study revealed a positive significant correlation between TLR2 and TLR4 expression and serum levels of kidney dysfunction biomarkers including cystatin C, creatinine, urea, and uric acid in diabetic patients with and without end-stage renal disease and kidney failure.

In agreement with our study, Dasu et al. (2010) found that the PB of patients with T2DM, which is related to the inflammatory reaction, has increased expression of TLR4. Similar results from 31 T1DM patients and 31 controls, Devaraj et al. (2008) examined TLR2 and TLR4 expression in monocytes. They found that the expression of TLR2 and TLR4 was significantly increased in T1DM monocytes compared with controls. Also, Dounousi et al. (2012) reported that CKD patients and patients with DNP are characterized by increased expression of TLRs 2 and 4 on monocytes. They measured expression of TLR2 and 4 in 37 CKD patients as group 1 (not having DM) and 19 CKD patients with DNP as group 2. Their results showed that group 1 patients exhibited only increased TLR2 membrane expression on monocytes compared with controls. Group 2 patients presented increased TLR2 and TLR4 membrane expression compared to the control group, and increased TLR4 expression compared to group 1. Kaur et al. (2012) reported increased TLR4 expression and activity under hyperglycemia in renal mesangial cells incriminating TLR4 in contributing to DNP. Lin et al. (2012) showed increased expression of TLR4 in human proximal tubular epithelial cells under hyperglycaemic conditions, pointing toward a role of TLR4 in tubulointerstitial inflammation in DNP. Also, our results agreed with the study of Mansour et al. (2014), which concluded that the level of TLR2 and 4 is elevated in patients with end-stage DNP compared to that of patients with end-stage renal disease due to non-DNP. Badr et al. (2019) found that TLRs were overexpressed in microvascular complicated type 2 diabetic patients as compared to non-complicated type 2 diabetic patients.

In the present study, higher expression of TLR2 and TLR4 in diabetic patients with kidney failure than in diabetic patients without kidney failure was associated with higher values of HOMA-IR (as indicator of insulin resistance) and lower values of HOMA-IS (as indicator of insulin sensitivity). In concomitance, the diabetic patients with end-stage renal disease also showed higher levels of serum inflammatory cytokines TNF-α, IL-6, and IFN-γ. These associations reflect the roles of TLR2 and TLR4 in the progress of insulin resistance and inflammation which in turn may lead the advance of nephrotic disease to the end-renal disease stage and renal failure (Figure 6). This suggestion is in concordance with the elucidation of Garibotto et al. (2017) who reported that TLR2 and TLR4 initiate inflammatory response and signaling for production of inflammatory cytokines that can induce insulin resistance and progress of DNP to the end-stage renal disease in T2DM. According to those later authors, it was proposed that the activation of TLRs stimulates the expression of several inflammatory cytokines and chemokines such as CCL2 IL-6 and TNF-α, which are associated with the progression of DNP (Figure 6). Moreover, Yiu et al. (2014) reported that TLR2 and TLR4 might play distinct roles in the pathogenesis of renal fibrosis; TLR2 initiates proinflammatory responses, whereas TLR4 mediates both proinflammatory and pro-fibrotic pathways.

As represented in schematic Figure 1, the stimulation of TLRs in T2DM by DAMPs and other ligands leads to the activation of the transcription nuclear factor NF-*κ*B, which is transferred to the nucleus and cleaved to p50 and p665 to induce inflammatory response and release of inflammatory cytokines and chemokines. The stimulation of TLR2 and TLR4 also leads to activation of activator protein-1 (AP-1), which also aggravates the inflammatory response through AP-1 target gene. The persistent increase in chemokines and inflammatory cytokines such as TNF-α, INF-γ, and IL-6 leads to progression of insulin resistance in one hand and of DNP to end-stage renal disease and kidney failure on the other.

In conclusion, TLR2 and TLR4 expressions are elevated in nephrotic diabetic patients with renal failure or without renal failure compared to normal individuals. TLR2 and TLR4 expressions are much more elevated in nephrotic diabetic patients with renal failure compared to nephrotic diabetic patients without renal failure. Moreover, TLR2 and TLR4 activity was markedly increased in association with elevated TNF-α, IL-6, and IFN-γ expressions, increased insulin resistance in response to inflammation; the changes were more prominent in diabetic patients with end-stage renal disease and renal failure. These observations significantly add to the emerging role of TLRs and Th1 inflammatory cytokines in the development and progress of DNP in type 2 diabetic patients. Moreover, it can be suggested that TLR2 and TLR4 may be a promising therapeutic targets to prevent or retard the DNP in type 2 diabetic patients.

## Data Availability Statement

The raw data supporting the conclusions of this article will be made available by the authors, without undue reservation.

## Ethics Statement

The studies involving human participants were reviewed and approved by Ethics Committee of Faculty of Post-graduate studies for Advanced Science, Beni-Suef University, Egypt. The patients/participants provided their written informed consent to participate in this study.

## Author Contributions

RA, AAh, WH, and OA designed the experiments. RA, AAh, WH, AR, TA, AAs, and OA conducted the experiments, analyzed the data, and edited and approved the final version of the manuscript. All authors contributed to the article and approved the submitted version.

### Conflict of Interest

The authors declare that the research was conducted in the absence of any commercial or financial relationships that could be construed as a potential conflict of interest.

## References

[ref1] Arababadi M. K. Naghavi N. Hassanshahi G. Mahmoodi M. (2009). Is CCR5-Delta32 mutation associated with diabetic nephropathy in type 2 diabetes? Ann. Saudi Med. 29:413. 10.4103/0256-4947.55177, PMID: 19700905 PMC3290048

[ref2] Badr R. E. Salama M. I. Abd-Elmaogood A. K. Eldeib A. E. M. (2019). Toll-like receptor 2 expression on monocytes and microvascular complications in type 2 diabetic patients. Diabetes Metab. Syndr. 13, 1299–1302. 10.1016/j.dsx.2019.01.038, PMID: 31336481

[ref3] Beisswenger P. J. Makita Z. Curphey T. J. Moore L. L. Jean S. Brinck-Johnsen T.. (1995). Formation of immunochemical advanced glycosylation end products precedes and correlates with early manifestations of renal and retinal disease in diabetes. Diabetes 44, 824–829. 10.2337/diab.44.7.824, PMID: 7789650

[ref4] Beutler B. (2004). Inferences, questions and possibilities in toll-like receptor signalling. Nature 430, 257–263. 10.1038/nature02761, PMID: 15241424

[ref5] Chang T. T. Chen J. W. (2020). The role of chemokines and chemokine receptors in diabetic nephropathy. Int. J. Mol. Sci. 21:3172. 10.3390/ijms21093172, PMID: 32365893 PMC7246426

[ref6] Chomczynski P. (1993). A reagent for the single-step simultaneous isolation of RNA, DNA and proteins from cell and tissue samples. Biotechniques 15, 532–537. PMID: 7692896

[ref7] Chomczynski P. Sacchi N. (1987). Single-step method of RNA isolation by acid guanidinium thiocyanate-phenol-chloroform extraction. Anal. Biochem. 162, 156–159. 10.1016/0003-2697(87)90021-2, PMID: 2440339

[ref8] Curtiss L. K. Tobias P. S. (2009). Emerging role of toll-like receptors in atherosclerosis. J. Lipid Res. 50, S340–S345. 10.1194/jlr.R800056-JLR200, PMID: 18980945 PMC2674724

[ref9] Dasu M. R. Devaraj S. Park S. Jialal I. (2010). Increased toll-like receptor (TLR) activation and TLR ligands in recently diagnosed type 2 diabetic subjects. Diabetes Care 33, 861–868. 10.2337/dc09-1799, PMID: 20067962 PMC2845042

[ref10] Devaraj S. Dasu M. R. Park S. H. Jialal I. (2009). Increased levels of ligands of toll-like receptors 2 and 4 in type 1 diabetes. Diabetologia 52, 1665–1668. 10.1007/s00125-009-1394-8, PMID: 19455302 PMC2709882

[ref11] Devaraj S. Dasu M. R. Rockwood J. Winter W. Griffen S. C. Jialal I. (2008). Increased toll-like receptor (TLR) 2 and TLR4 expression in monocytes from patients with type 1 diabetes: further evidence of a proinflammatory state. J. Clin. Endocrinol. Metab. 93, 578–583. 10.1210/jc.2007-2185, PMID: 18029454 PMC2243229

[ref72] Dounousi E. Koliousi E. Papagianni A. Ioannou K. Zikou X. Katopodis K.. (2012). Mononuclear leukocyte apoptosis and inflammatory markers in patients with chronic kidney disease. Am. J. Nephrol. 36, 531–536. 10.1159/000345352, PMID: 23258075

[ref12] Fawcett J. K. Scott J. E. (1960). A rapid and precise method for the determination of urea. J. Clin. Pathol. 13, 156–159. 10.1136/jcp.13.2.156, PMID: 13821779 PMC480024

[ref15] Garibotto G. Carta A. Picciotto D. Viazzi F. Verzola D. (2017). Toll-like receptor-4 signaling mediates inflammation and tissue injury in diabetic nephropathy. J. Nephrol. 30, 719–727. 10.1007/s40620-017-0432-8, PMID: 28933050

[ref18] Gochman N. Schmitz J. M. (1971). Automated determination of uric acid, with use of a uricase-peroxidase system. Clin. Chem. 17, 1154–1159. 10.1093/clinchem/17.12.1154, PMID: 5118153

[ref21] Grant S. F. Thorleifsson G. Reynisdottir I. Benediktsson R. Manolescu A. Sainz J.. (2006). Variant of transcription factor 7-like 2 (TCF7L2) gene confers risk of type 2 diabetes. Nat. Genet. 38, 320–323. 10.1038/ng1732, PMID: 16415884

[ref22] Helou C. Marier D. Jacolot P. Abdennebi-Najar L. Niquet-Léridon C. Tessier F. J.. (2014). Microorganisms and Maillard reaction products: a review of the literature and recent findings. Amino Acids 46, 267–277. 10.1007/s00726-013-1496-y, PMID: 23588491

[ref24] Jaffé M. (1886). Ueber den Niederschlag, welchen Pikrinsäure in normalem Harn erzeugt und über eine neue Reaction des Kreatinins. Z. Physiol. Chem. 10, 391–400.

[ref25] Jialal I. Kaur H. (2012). The role of toll-like receptors in diabetes-induced inflammation: implications for vascular complications. Curr. Diab. Rep. 12, 172–179. 10.1007/s11892-012-0258-7, PMID: 22314791

[ref27] Kaur H. Chien A. Jialal I. (2012). Hyperglycemia induces toll like receptor 4 expression and activity in mouse mesangial cells: relevance to diabetic nephropathy. Am. J. Physiol. Ren. Physiol. 303, F1145–F1150. 10.1152/ajprenal.00319.2012, PMID: 22874765 PMC3469679

[ref30] Kindmark C. O. (1972). The concentration of C-reactive protein in sera from healthy individuals. Scand. J. Clin. Lab. Invest. 29, 407–411. 10.3109/00365517209080258, PMID: 21488409

[ref31] Krishnaveni G. V. Veena S. R. Hill J. C. Kehoe S. Karat S. C. Fall C. H. (2010). Intrauterine exposure to maternal diabetes is associated with higher adiposity and insulin resistance and clustering of cardiovascular risk markers in Indian children. Diabetes Care 33, 402–404. 10.2337/dc09-1393, PMID: 19918007 PMC2809291

[ref32] Kuang L. Huang Z. Hong Z. Chen A. Li Y. (2015). Predictability of 1-h postload plasma glucose concentration: a 10-year retrospective cohort study. J. Diabetes Investig. 6, 647–654. 10.1111/jdi.12353, PMID: 26543538 PMC4627541

[ref33] Lepenies J. Eardley K. S. Kienitz T. Hewison M. Ihl T. Stewart P. M.. (2011). Renal TLR4 mRNA expression correlates with inflammatory marker MCP-1 and profibrotic molecule TGF-beta(1) in patients with chronic kidney disease. Nephron Clin. Pract. 119, c97–c104. 10.1159/000324765, PMID: 21677444

[ref34] Levey A. S. Bosch J. P. Lewis J. B. Greene T. Rogers N. Roth D. (1999). A more accurate method to estimate glomerular filtration rate from serum creatinine: a new prediction equation. Modification of diet in renal disease study group. Ann. Intern. Med. 130, 461–470. 10.7326/0003-4819-130-6-199903160-00002, PMID: 10075613

[ref35] Levey A. S. Coresh J. Greene T. Stevens L. A. Zhang Y. L. Hendriksen S.. (2006). Using standardized serum creatinine values in the modification of diet in renal disease study equation for estimating glomerular filtration rate. Ann. Intern. Med. 145, 247–254. 10.7326/0003-4819-145-4-200608150-00004, PMID: 16908915

[ref36] Lim A. K. Tesch G. H. (2012). Inflammation in diabetic nephropathy. Mediat. Inflamm. 2012:146154. 10.1155/2012/14615, PMID: 22969168 PMC3432398

[ref37] Lin M. Yiu W. H. Wu H. J. Chan L. Y. Leung J. C. Au W. S.. (2012). Toll-like receptor 4 promotes tubular inflammation in diabetic nephropathy. J. Am. Soc. Nephrol. 23, 86–102. 10.1681/ASN.2010111210, PMID: 22021706 PMC3269929

[ref39] Liu K. H. Zhou Q. L. Ao X. Tang T. F. Hong X. M. Bao R. L. (2010). Effect of spironolactone on the expression of toll-like receptor 4 in renal tubular epithelia cells exposed to high glucose. Chin. J. Contemp. Pediatr. 12, 280–283. PMID: 20416221

[ref40] Mansour M. Salam R. F. Rashed L. Salam H. (2014). Role of toll receptors in diabetic nephropathy. J. Diabetes Mellit. 4, 26–32. 10.4236/jdm.2014.41005

[ref42] McCance D. R. Dyer D. G. Dunn J. A. Bailie K. E. Thorpe S. R. Baynes J. W.. (1993). Maillard reaction products and their relation to complications in insulin-dependent diabetes mellitus. J. Clin. Invest. 91, 2470–2478. 10.1172/JCI116482, PMID: 8514859 PMC443307

[ref44] Mishra V. Pathak C. (2019). Human toll-like receptor 4 (hTLR4): structural and functional dynamics in cancer. Int. J. Biol. Macromol. 122, 425–451. 10.1016/j.ijbiomac.2018.10.142, PMID: 30365988

[ref46] Navarro-Gonzalez J. F. Mora-Fernandez C. (2008). The role of inflammatory cytokines in diabetic nephropathy. J. Am. Soc. Nephrol. 19, 433–442. 10.1681/ASN.2007091048, PMID: 18256353

[ref47] Nosratabadi R. Arababadi M. K. Hassanshahi G. Yaghini N. Pooladvand V. Shamsizadeh A.. (2009). Evaluation of IFN-gamma serum level in nephropatic type 2 diabetic patients. Pak. J. Biol. Sci. 12, 746–749. 10.3923/pjbs.2009.746.749, PMID: 19634484

[ref48] Otsuji S. Shibata H. Umeda M. (1982). Turbidimetric immunoassay of serum C-reactive protein. Clin. Chem. 28, 2121–2124. 10.1093/clinchem/28.10.2121, PMID: 6812987

[ref49] Panchapakesan U. Pollock C. (2018). The role of toll-like receptors in diabetic kidney disease. Curr. Opin. Nephrol. Hypertens. 27, 30–34. 10.1097/MNH.0000000000000377, PMID: 29059081

[ref50] Park J. M. Bong H. Y. Jeong H. I. Kim Y. K. Kim J. Y. Kwon O. (2009). Postprandial hypoglycemic effect of mulberry leaf in Goto-Kakizaki rats and counterpart control Wistar rats. Nutr. Res. Pract. 3, 272–278. 10.4162/nrp.2009.3.4.272, PMID: 20098579 PMC2809233

[ref51] Pashkow F. J. (2011). Oxidative stress and inflammation in heart disease: do antioxidants have a role in treatment and/or prevention? Int. J. Inflamm. 2011:514623. 10.4061/2011/514623, PMID: 21860805 PMC3157078

[ref52] Pérez-Morales R. E. del Pino M. D. Valdivielso J. M. Ortiz A. Mora-Fernández C. Navarro-Gonzáleza J. F. (2019). Inflammation in diabetic kidney disease. Nephron 143, 12–16. 10.1159/000493278, PMID: 30273931

[ref55] Rayego-Mateos S. Morgado-Pascual J. L. Opazo-Ríos L. Guerrero-Hue M. García-Caballero C. Vázquez-Carballo C.. (2020). Pathogenic pathways and therapeutic approaches targeting inflammation in diabetic nephropathy. Int. J. Mol. Sci. 21:3798. 10.3390/ijms21113798, PMID: 32471207 PMC7312633

[ref71] Rosa R. S. Ravi K. D. M. (2012). Toll-like receptors and diabetes complications: recent advances. Curr. Diabetes Rev. 8, 480–488. 10.2174/157339912803529887, PMID: 22934553

[ref56] Ruiz-Ortega M. Rodrigues-Diez R. R. Lavoz C. Rayego-Mateos S. (2020). Editorial: special issue “diabetic nephropathy: diagnosis, prevention and treatment.” J. Clin. Med. 9:813. 10.3390/jcm9030813, PMID: 32192024 PMC7141346

[ref57] Ruster C. Wolf G. (2008). The role of chemokines and chemokine receptors in diabetic nephropathy. Front. Biosci. 13, 944–955. 10.2741/2734, PMID: 17981602

[ref58] Shi H. Kokoeva M. V. Inouye K. Tzameli I. Yin H. Flier J. S. (2006). TLR4 links innate immunity and fatty acid-induced insulin resistance. J. Clin. Invest. 116, 3015–3025. 10.1172/JCI28898, PMID: 17053832 PMC1616196

[ref59] Singh V. P. Bali A. Singh N. Jaggi A. S. (2014). Advanced glycation end products and diabetic complications. Korean J. Physiol. Pharmacol. 18, 1–14. 10.4196/kjpp.2014.18.1.1, PMID: 24634591 PMC3951818

[ref61] Tang S. C. Yiu W. H. (2020). Innate immunity in diabetic kidney disease. Nat. Rev. Nephrol. 16, 206–222. 10.1038/s41581-019-0234-4, PMID: 31942046

[ref62] Trinder P. (1969). Determination of blood glucose using 4-amino phenazone as oxygen acceptor. J. Clin. Pathol. 22:246. 10.1136/jcp.22.2.246-b, PMID: 5776563 PMC474047

[ref63] White S. L. Cass A. Atkins R. C. Chadban S. J. (2005). Chronic kidney disease in the general population. Adv. Chronic Kidney Dis. 12, 5–13. 10.1053/j.ackd.2004.10.009, PMID: 15719328

[ref64] Wong C. K. Ho A. W. Y. Tong P. C. Y. Yeung C. Y. Kong A. P. S. Lun S. W. M.. (2007). Aberrant activation profile of cytokines and mitogen-activated protein kinases in type 2 diabetic patients with nephropathy. Clin. Exp. Immunol. 149, 123–131. 10.1111/j.1365-2249.2007.03389.x, PMID: 17425653 PMC1942021

[ref65] Xu J. Chen L. J. Yu J. Wang H. J. Zhang F. Liu Q.. (2018). Involvement of advanced glycation end products in the pathogenesis of diabetic retinopathy. Cell. Physiol. Biochem. 48, 705–717. 10.1159/000491897, PMID: 30025404

[ref66] Yang J. Zhou Y. Guan Y. (2012). PPARγ as a therapeutic target in diabetic nephropathy and other renal diseases. Curr. Opin. Nephrol. Hypertens. 21, 97–105. 10.1097/MNH.0b013e32834de526, PMID: 22143250

[ref67] Yiu W. H. Lin M. Tang S. C. (2014). Toll-like receptor activation: from renal inflammation to fibrosis. Kidney Int. Suppl. 4, 20–25. 10.1038/kisup.2014.5, PMID: 26312146 PMC4536963

[ref68] Yonekura H. Yamamoto Y. Sakurai S. Watanabe T. Yamamoto H. (2005). Roles of the receptor for advanced glycation endproducts in diabetes-induced vascular injury. J. Pharmacol. Sci. 97, 305–311. 10.1254/jphs.CPJ04005X, PMID: 15750291

[ref69] Zhang X. Kong J. Yun K. (2020). Prevalence of diabetic nephropathy among patients with type 2 diabetes mellitus in China: a meta-analysis of observational studies. J. Diabetes Res. 2020:2315607. 10.1155/2020/2315607, PMID: 32090116 PMC7023800

[ref70] Zhu N. Zhou Y. Yuan W. J. Liu J. Shang M. H. Wang L.. (2011). Toll-like receptor 4 deposition and its significance in hepatitis B virus associated nephropathy. Zhonghua Nei Ke Za Zhi 50, 1008–1012. PMID: 22333167

